# Effect of Crisis Counselling on the Anxiety of Women With an Unplanned Pregnancy

**DOI:** 10.1155/jp/6682179

**Published:** 2026-02-06

**Authors:** Sabura Faqhani, Forouzan Elyasi, Seyed Abolhassan Naqibi, Seyed Nouroldin Mousavi Nasab, Mohammad Geran, Soghra Khani

**Affiliations:** ^1^ Student Research Committee, Mazandaran University of Medical Sciences, Sari, Iran, mazums.ac.ir; ^2^ Psychiatry and Behavioral Sciences Research Center, School of Medicine, Mazandaran University of Medical Sciences, Sari, Iran, mazums.ac.ir; ^3^ Health Services Management and Biostatistics, School of Health, Mazandaran University of Medical Sciences, Sari, Iran, mazums.ac.ir; ^4^ Health Sciences Research Center, School of Health, Mazandran University of Medical Sciences, Sari, Iran; ^5^ Qaemshahr Health Network, Mazandaran University of Medical Sciences, Sari, Iran, mazums.ac.ir; ^6^ Department of Reproductive Health and Midwifery Sexual and Reproductive Health Research Center, Faculty of Nursing and Midwifery, Sari, Iran

**Keywords:** crisis intervention, unplanned pregnancy, unwanted pregnancy

## Abstract

Pregnancy is one of the primary sources of stress, and unplanned pregnancy is a crisis in women′s lives. In this study, we sought to determine the effect of crisis counseling on women′s anxiety with an unplanned pregnancy. This semi‐experimental study was conducted using the convenience sampling method. Sixty married pregnant women aged 15–49 years with unplanned pregnancies were randomly assigned to the two groups of intervention and control (*n* = 30 per group), of whom five were excluded from the intervention group, and three were excluded from the control group. The data collection tools included a medical–mental health checklist, the 28‐item General Health Questionnaire‐28 (GHQ‐28), Winfield and Taigman′s Social Support Scale, and Spielberg′s State–Trait Anxiety Inventory (STAI). Three individual counseling sessions were held according to Roberts′ seven‐stage model. The participants′ state and trait anxiety scores were assessed before and one month after the counseling sessions. There was no significant difference between the two groups regarding demographic characteristics, general health score, level of social support, and the mean score of general anxiety (*p* > 0.05). The mean scores of state anxiety before and after the intervention in the interventional group were 45.92 ± 6.8 and 42.8 ± 4.14, respectively, and the mean of trait anxiety scores were 46.84 ± 6.82 and 44.48 ± 5.46, respectively. In the control group, the mean of state anxiety scores before and after the intervention were 46.78 ± 6.09 and 46.63 ± 7.1, respectively, and the mean of trait anxiety scores were 46.41 ± 4.54 and 46.89 ± 5.09, respectively. Crisis counseling significantly impacted both state (*p* = 0.004) and trait (*p* = 0.047) anxiety. Crisis counseling reduces trait and state anxiety in women with an unplanned pregnancy. Therefore, establishing high‐risk pregnancy clinics and employing midwifery consultants to assess and reduce anxiety levels in women with unplanned pregnancies will be beneficial.

**Trial Registration:**

Iranian Registry of Clinical Trials:IRCT2017100231117N5

## 1. Introduction

Unplanned pregnancy (unwanted and mistimed) refers to a pregnancy in which the couple has not planned [1, 2] or has planned for this occurrence in the future so that the current pregnancy is considered mistimed [3]. In other words, an unplanned pregnancy occurs when one or both parents do not wish for it [1]. Almost half of all pregnancies in the United States are unplanned [4, 5]. The ratio of wanted to unwanted pregnancies in Asia is 49–78 per 1,000 women of childbearing age [6]. In 2014, the prevalence of unwanted pregnancy in Iran was reported to be 30.6% [7].

Unwanted pregnancy affects two critical health indicators: maternal and child mortality [6]. Unwanted pregnancy leads to health, social, and economic problems [8] and imposes high costs on health systems [1, 6, 9, 10]. The side effects of unwanted pregnancy include low birth weight (LBW), preterm delivery, insufficient prenatal care, and increased risk of postpartum depression and psychological distress [11]. In 2012, approximately 40% of all pregnancies were unintended, and 50%, 13%, and 38% resulted in miscarriage, fetal death, and premature birth, respectively [8, 12].

Pregnancy is associated with higher rates of certain common anxiety disorders, such as generalized anxiety disorder [13], and unwanted pregnancy is a crisis in women′s lives [14]. Clinically, half of women experience significant stress levels during pregnancy [4, 5, 15], making them vulnerable to mental pressure. Women with unwanted pregnancies are more likely to need psychosocial assessment and/or coordinated care to cope with unwanted pregnancies until delivery [16].

A crisis is an acute disturbance in the mental balance due to which the normal coping mechanism fails, and evidence of distress and dysfunction is discernible [17]. The types of interventions to deal with unwanted pregnancy crises are options counseling [14], medical crisis counseling [18], and mandatory counseling [19]. Options counseling is also a crisis intervention beneficial for women who know they are pregnant and must be clear about their feelings and thoughts about different options [5]. This strategy promotes commitment to women′s independence and dignity and supports women in their choices [14]. In medical crisis counseling, patients′ medical concerns are the main focus of intervention [18]. Mandatory counseling and waiting period, despite lowering the rate of abortion, increases the number of second‐trimester abortions, neglects autonomy, causes frequent visits to the clinic, and causes physical and psychological distress [14].

Crisis intervention is appropriate immediately after a crisis and is short‐lived, lasting only one to six weeks [20]. The counseling leads to a decrease in the use of avoidance strategies among women facing unplanned pregnancies [21], and it may allow women in crisis to choose other alternatives to abortion [22]. Appropriate crisis interventions prevent mental disorders′ early or long‐term symptoms and promote individual prosperity and growth [23]. Various studies have been performed on crisis intervention for accidents [24] and natural disasters [25], war [26, 27], trauma [28], mental illness, atopic dermatitis [29], and family members of patients in a vegetative state [30].

Although all of the mentioned intervention models were designed to help resolve an unwanted pregnancy crisis, none are designed and implemented to decrease anxiety and return a person to the precrisis levels of function. Additionally, interventional studies among women with unwanted pregnancies were performed only in non‐Iranian contexts and did not aim at reducing anxiety. Due to the severity and complications of unwanted pregnancy crises, this study was conducted to determine the effect of crisis counseling on the level of anxiety in women with unplanned pregnancies.

## 2. Methods

This semi‐experimental study was performed using a pretest–posttest control‐group design. It was approved by the Research Ethics Committee of Mazandaran University of Medical Sciences (code: IR.mazums.rec.1396.3181). The statistical population included married women of childbearing age (15–49 years). Sampling was performed using the convenience sampling method. The sample size was estimated using the following formula and the number of samples required in covariance analysis studies [31]. In this formula, according to the study of Khanzadeh et al. [32], the standard deviation of both groups is considered 55, and the minimum difference between the two groups is 40 units. With *α* = 0.05 and 1 − *β* = 0.90 and a correlation coefficient of 0.50, the standard sample size was obtained for 30 people in each group.

n=Z12−α+Z−β2σ12+σ22μ1−μ22×1−ρ2,n=1.961.29+2552+552402 ×10.5−2.

The inclusion criteria included women of childbearing age; having at least one child; Iranian nationality; living with a legal spouse; ability to communicate; having physical health such as not having diabetes (according to the person), hypertension, any known chronic diseases such as cardiovascular, renal, or hepatic diseases [33]; and a positive pregnancy test (*β*‐hCG). The exclusion criteria comprised having major and acute psychiatric disorders, psychosis (the patient was referred to a psychiatrist if these symptoms were present), participation in other psychiatric treatment protocols in the last six months, and unwillingness to participate in the study [34].

Sampling was performed in 18 urban community health centers, 82 gynecology offices, and 7 midwifery offices in Qaemshahr, Iran, from November 2017 until August 2018. First, the city′s list of urban community health centers, gynecology offices, and midwifery offices was prepared. In a briefing session, doctors and midwives working in these centers provided the necessary explanations on how to carry out the project. The eligible women were asked to be introduced to the student who implemented the project. People visiting these centers to request a pregnancy test and who did not intend to become pregnant, and those who did not intend to become pregnant but had a positive pregnancy test in the past four weeks and visited the center for registration, were invited to participate in the project, and their phone numbers were given to the project manager.

After obtaining informed consent, a demographics checklist, a medical history checklist, and a general health questionnaire were completed. If the inclusion criteria were met, the participants were randomly assigned to one of the two groups. In detail, using Random Number Generator software, two blocks of 35 random numbers were obtained, and consecutive patients were assigned to the groups based on these numbers. Afterward, Winfield and Taigman′s Social Support Scale and Spielberg′s State–Trait Anxiety Inventory (STAI) were completed by participants. After coordination with the participants in the intervention group, according to the schedule, the participants were asked to be present at a specific place at an appointed time, and counseling was provided. The first session was held as soon as possible, four weeks after the pregnancy test. Three individual counseling sessions of 120–150 min with an interval of three or four days were held. This study provided counseling to the intervention group based on Roberts′ seven‐stage model. The control group also received routine pregnancy care. The two groups were reevaluated for anxiety one month after completing the counseling program.

### 2.1. Data Collection Tools

The demographic checklist included 17 items. The General Health Questionnaire‐28 (GHQ‐28) contains 28 questions about a person′s general health status in the past month. The GHQ‐28 was designed by Goldberg and Hiller [35] and has four subscales, each including seven questions. The mentioned subscales are (1) the Somatic Symptoms subscale, (2) Anxiety and Sleep Disorders subscale, (3) the Social Functions subscale, and (4) the Depression Symptoms subscale. In some studies, the reliability of the questionnaire has been established using the retest method.

STAI consists of 40 questions, of which 20 are related to state anxiety, and 20 are related to trait anxiety. The items are rated on a 4‐point Likert scale, and the possible scores range from 20 to 80. State anxiety measures anxiety over some time, in which the answer to each question is in the form of four options (i.e., *basically*, *partially*, *moderately*, and *very much*). Still, trait anxiety measures the general and permanent feeling. It shows an anxious personality and has four‐choice answers (i.e., *almost never*, *almost sometimes*, *most of the time*, and *almost always*).

Winfield and Taigman′s Social Support Scale consists of 16 questions in two parts; the first 10 questions have four choices, and the next six questions regard postpartum social support. Each question of the first part is given a score of 0–4, and the total score can range from 0 to 40 points. A score of 0–10 is considered low social support; a score of 11–20 is considered moderate, and a score above 21 is high social support. Each question in the second part is scored 0 or 1.

### 2.2. Intervention Description

The main purpose of the counseling sessions was to encourage the participants to be aware of their conflicting feelings and struggles, thus helping them to understand and resolve these anxiety‐inducing conflicts. The ultimate goal was to return to precrisis function. The sessions were both informational and process‐oriented, and the women were allowed to express what might evoke unacceptable feelings of anger, guilt, anxiety, depression, jealousy, hatred, and so on. The main focus was on anxiety and feelings of control and self‐threat. The treatment program for the interventional group was presented based on Roberts′ seven‐stage model. The contents of the sessions are shown in Table 1.

**Table 1:** Content of the sessions according to Roberts′ seven‐step counseling model.

**Session**	**Contents**
First session	Introduction: completing questionnaires and holding pretests; establishing cooperative therapeutic communication; examining main and major problems including crisis emergencies; assessing the level of immediate risk for clients and others, the likelihood of self‐harming behavior, individual history, and current status of mental health function (anger, hatred, depression, suicide, anxiety, violence, and hallucinations)
Second session	Encouraging to explore feelings, discover and evaluating previous compromising strategies
Third session	Restoring the executive function of the individual through the implementation of a performance plan based on problem‐solving methods and various decision‐making models
One month later	Reviewing the entire program, explaining about maintaining the achievements and preventing the return of the signs, and at the end, the posttest.

### 2.3. Statistical Analysis

First, based on the Kolmogorov–Smirnov test, the data was confirmed to be normal, and then descriptive statistics (frequency distribution table, mean, and standard deviation) were used to describe the demographic characteristics. Next, a *t*‐test was used for dichotomous variables and ANOVA was run for categorical variables to determine the related variables. An independent *t*‐ test (between intervention and control groups) and paired *t*‐test (in each group before and after the intervention) were run to assess the effect of crisis counseling on anxiety. A *p* value of less than 0.05 was considered significant. Data analysis was performed using SPSS Version 21.

## 3. Results

**Figure 1:**
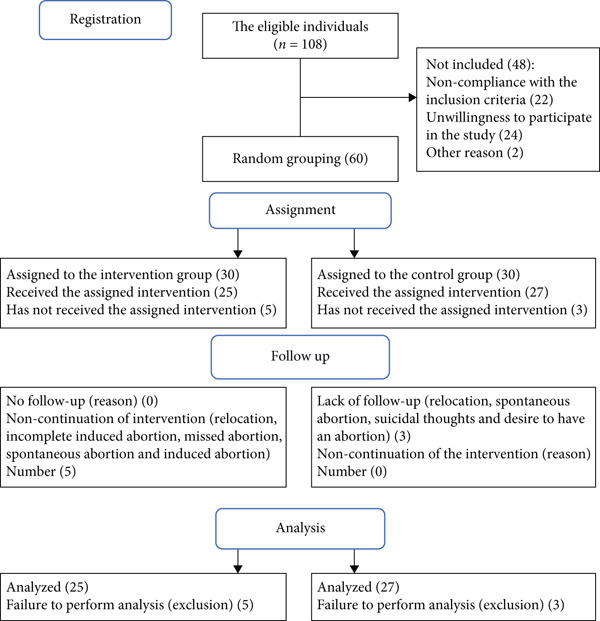
Consort flow chart of the study.

Out of the 108 individuals, 60 women met the inclusion criteria and were randomly divided into the two groups of intervention and control (*n* = 30 per group). Data related to five patients in the intervention group and three patients in the control group were excluded from the statistical analyses after the intervention due to the exclusion criteria. Overall, 52 participants were enrolled in the study. Both intention‐to‐treat and perprotocol approaches were used to evaluate the effect of sample attrition (Figure 1).

The distribution of variables in the two groups for the anxiety score is normal and is not significantly different (*p* > 0.05). Also, the distribution of variables in the two groups for the trait anxiety score was not significantly different (*p* > 0.05).

Our findings showed the two groups were homogeneous in terms of age, parity, the use of contraceptives, the use of emergency method in the last unprotected intercourse, awareness of ovulation time, the use of emergency method in the last menstrual cycle, type of pregnancy (unwanted for the mother, unwanted for both parents), previous marriage, number of children from previous marriage, history of physical and mental illnesses, occurrence of stressful events during the past year, age of marriage, level of education (couple), occupational status (husband and wife), number of household members, household income, socioeconomic status (Table 2), and level of social support (Table 3). Also, the two groups were similar in terms of all the dimensions of general health and the total score of this index during the last month (Table 4). Nevertheless, they were not homogeneous regarding the history of abortion (*p* < 0.05) (Table 2).

**Table 2:** Comparison of the intervention and control groups in terms of demographic characteristics.

**Variable**	**Intervention** **(** **n** = 30**)**	**Control** **(** **n** = 30**)**	**p**
Wife age ^∗^ (years)	30.9 (5.4)	30.6 (5.9)	0.252
Husband age ^∗^	35.6 (5.3)	34. 4 (6.1)	0.815
Gestational age (weeks) ^∗^	6.3 (2.4)	7.5 (3.7)	0.449
Sampling location ^∗∗^			
Hospital prenatal clinic	14 (46.7)	15 (50)	0.139
Urban family physician	11 (36.7)	5 (16.7)	
Location ^∗∗^			
City	23 (76.7)	19 (50.0)	0.26
Village	7 (23.3)	11 (35.5)	
Wife education ^∗∗^			
Primary	5 (16.7)	7 (23.3)	0.714
Diploma and postdiploma	18 (60.0)	15 (50.0)	
Bachelor′s degree and higher	7 (23.3)	8 (26.7)	
Husband education ^∗∗^			
Primary	9 (30.0)	13 (43.3)	0.406
Diploma and postdiploma	15 (50.0)	10 (32.3)	
Bachelor′s degree and higher	6 (20.0)	7 (25.8)	
Female employment ^∗∗^			
Employed	26 (86.7)	28 (93.3)	0.38
Housewife	4 (13.3)	2 (6.7)	
Husband employment ^∗∗^			
Employed	30 (100)	28 (94.5)	
Unemployed	0	2 (5.5)	0.15
Adequacy of household income ^∗∗^			
Totally enough	6 (20.0)	3 (10.0)	
It is somewhat sufficient	20 (66.7)	20 (64.5)	0.403
It is not enough at all	4 (13.3)	7 (22.6)	
Socioeconomic class ^∗∗^			
Top	2 (6.7)	1 (3.3)	
Middle	23 (76.7)	22 (73.3)	0.709
Low	5 (16.7)	7 (23.3)	
No			
Difficulty in caring for the baby ^∗∗^	15 (50.0)	11 (36.7) 19 (61.3)	0.297
Yes	15 (50.0)		
No			
Type of pregnancy ^∗∗^	6 (20.0)	4 (13.3)	0.488
Unwanted for woman	24 (80.0)	26 (86.7)	
Unwanted for both			
History of miscarriage ^∗∗^	20 (66.7)	27 (90.3)	0.028
No	10 (33.3)	3 (10.0)	
Yes			
History of abortion ^∗∗^	25 (83.3)	29 (96.8)	0.043
No	5 (16.7)	1 (3.3)	
Yes			
Interval of two recent pregnancies < 2 years ^∗∗^	11 (63.7)	9 (30.0)	0.584
No	19 (63.3)	21 (70.0)	
Yes			
Action to terminate pregnancy ^∗∗^	5 (16.7)	8 (26.7)	0.347
Yes	25 (83.3)	22 (73.3)	
No			

^∗^ Mean (standard deviation), *t*‐test or ANOVA.

^∗∗^ Number (frequency percentage), chi‐square.

**Table 3:** Comparison of experimental and control groups in terms of social support score.

**Variable**	**Intervention** **(** **n** = 30**)**	**Control** **(** **n** = 30**)**	**p** ^∗^
Social support score	26.30 (6.09)	26.0 (5.45)	0.652
General health score	4.70 (1.37)	4.57 (1.88)	0.244

^∗^ Mean (standard deviation), *t*‐test.

**Table 4:** Comparison of experimental and control groups in terms of general health score (28‐item GHQ‐28).

**Variable**	**Intervention** **(** **n** = 30**)**	**Control** **(** **n** = 30**)**	**p** ^∗^
Somatic Symptoms scale	4.53 (3.27)	4.07 (4.79)	0.089
Anxiety and Sleep Disorders scale	6.63 (4.22)	4.73 (4.38)	0.968
Social Function scale	8.93 (2.27)	8.07 (3.10)	0.271
Depression Symptoms scale	3.63 (3.10)	2.43 (4.02)	0.758
General health score	23.7 (9.26)	19.30 (13.86)	0.144

^∗^ Mean (standard deviation), *t*‐test.

The results of this study revealed that with the perprotocol method, the mean score of state anxiety in the pre‐intervention stage was 47.43 ± 6.59 in the intervention group and 47.9 ± 6.35 in the control group. There was no significant difference between the two groups at the pre‐intervention stage (*p* = 0.114). At the postintervention stage, the mean state anxiety score was 43.53 ± 3.22 in the intervention group and 46.11 ± 6.97 in the control group. The mean scores of the two groups in the postintervention phase were significantly different (*p* = 0.037). The difference in the mean scores of state anxiety between the two groups before and after the intervention was also significantly different (*p* = 0.005) (Table 5).

**Table 5:** Anxiety score and status of the studied samples (with preprotocol approach).

**Variable**	**Intervention** **(** **n** = 30**)**	**Control** **(** **n** = 30**)**	**p**
State Anxiety			
Pre‐intervention ^∗^	47.03 (6.59)	47.9 (6.35)	0.114
Postintervention ^∗^	43.53 (3.22)	46.11 (6.97)	0.037
*p* value ^∗∗^	0.02 ^∗∗^	0.386 ^∗∗^	—
Difference	−3.5 (4.0)	−1.79 (2.15)	0.005
Trait anxiety ^∗^			
Pre‐intervention ^∗^	46.30 (6.4)	46.53 (5.3)	0.548
Postintervention ^∗^	44.66 (4.99)	46.69 (4.86)	0.064
*p* value ^∗∗^	0.239 ^∗∗^	0.857 ^∗∗^	—
Difference ^∗^	−2.35 (4.8)	0.16 (4.51)	0.046

^∗^ Mean (standard deviation), *t*‐test.

^∗∗^Wilcoxon signed test.

The mean score of trait anxiety in the pre‐intervention stage was 46.30 ± 6.37 in the intervention group and 46.53 ± 5.3 in the control group. Therefore, the anxiety scores of the two groups were not significantly different at the pre‐intervention stage (*p* = 0.548). At the postintervention stage, the mean score was 44.66 ± 4.99 in the intervention group and 46.69 ± 4.86 in the control group. There was a significant difference in the mean trait anxiety score within the two groups before and after the intervention (*p* = 0.046). However, the mean trait anxiety score of the two groups in the postintervention phase was not significantly different (*p* = 0.064) (Table 5).

With the intention‐to‐treat method (five patients in the intervention group and three patients in the control group did not participate in the second round of evaluation), the mean score of state anxiety at the pre‐intervention stage in the interventional group was 45.92 ± 6.8. In the control group, it was 46.78 ± 6.09. The two groups had no significant difference regarding state anxiety scores at the pre‐intervention stage (*p* = 0.286). The difference in the mean state anxiety score in the two groups was significantly different at the pre‐intervention and postintervention stages (*p* = 0.017). The mean score of trait anxiety at the postintervention stage was 46.84 ± 6.8 in the interventional group, and in the control group, it was 46.41 ± 4.54. The two groups had a significant difference regarding the mean trait anxiety score at the postintervention stage (*p* = 0.032). There was a considerable difference in the mean trait anxiety score between the two groups at the pre‐intervention and postintervention stages (*p* = 0.041) (Table 6).

**Table 6:** Anxiety score of state and trait of the studied samples (with the intention‐to‐treat approach).

**Variable**	**Intervention** **(** **n** = 25**)**	**Control** **(** **n** = 27**)**	**p** ^∗^
State anxiety			
Pre‐intervention	45.92 (6.8)	46.78 (6.09)	0.286
Postintervention	42.8 (4.14)	46.63 (7.1)	0.04
*p* value ^∗∗^	0.017	0.161	—
Difference	−3.12 (5.2)	−0.15 (2.77)	0.004
Trait anxiety			
Pre‐intervention	46.84 (6.8)	46.41 (4.54)	0.788
Postintervention	44.48 (5.463)	46.86 (5.09)	0.032
*p* value ^∗∗^	0.041	0.936	—
Difference	−2.36 (5.27)	0.482 (4.76)	0.047

^∗^ Mean (standard deviation), *t*‐test.

^∗∗^Wilcoxon signed test.

## 4. Discussion

The results showed crisis counseling caused a significant difference in the trait anxiety level (state anxiety and trait anxiety) of pregnant women with unplanned pregnancies. Although there is a lack of studies showing the effect of this type of counseling on the level of anxiety in women with an unplanned pregnancy, the results of this study were in line with those of several studies on the effectiveness of various methods of anxiety reduction, such as spiritual intelligence training [36], midwifery counseling program [37, 38], cognitive–behavioral training [32], and group therapy for stress management.

A study by Gordon [38] on the effect of crisis counseling on trait anxiety in men who accompanied women for abortion showed that a 2‐h group crisis counseling session reduced state anxiety in the group receiving counseling. Still, it did not affect trait anxiety [38]. The results of the present study contradict Gordon′s findings regarding trait anxiety. The difference in conclusions is justifiable because Gordon′s study was performed among men and because the interval between the initial and postintervention evaluations was different in the two studies. In the mentioned study, a 1‐month interval between the two assessments increased the long‐term effects of counseling and showed reduced trait anxiety in people receiving counseling. Gordon′s study exhibited that group counseling is an effective tool to help cope with the personal crises of men whose wives seek legal abortion. The effectiveness of such short‐term counseling sessions may, at least in part, increase a person′s sensitivity to psychological help during a crisis. Overall, the group crisis counseling approach based on the community mental health crisis intervention theory is an effective tool to help a formally psychologically overlooked population [38].

This study′s results align with the findings of Ross‐Reynolds and Hardy [39], who examined the effect of crisis counseling on adolescent sexual disorders related to pregnancy and homosexuality. In Ross‐Reynolds and Hardy′s study, a psychologist assumed the role of a crisis counselor to facilitate the rapid reduction of acute stress, improve adolescents′ coping skills, and help them access their personal and social resources. Whether it was pregnancy or homosexuality, the psychologist accepted the adolescent exposed to the crisis and created an environment where they could feel safe and talk about their feelings and concerns [39].

It seems that one of the reasons for the effectiveness of this type of counseling can be that the counseling was performed individually in a peaceful and confidential environment, and the participants felt safe in expressing their feelings and personal issues. They also had ample opportunity to discuss their feelings and emotions about the pregnancy. Providing counseling in several separate sessions with an interval of three or four days made the participants think about the issues raised in each session, do the exercises related to each session, and better understand their feelings about the current pregnancy.

### 4.1. Limitations of the Study

Since unplanned pregnancy, especially unwanted pregnancy, causes high levels of anxiety in the affected people and, in the long run, makes the possibility of therapeutic interventions more complicated, most of them implement their decision as soon as possible. According to Articles 622 and 623 of the Constitution of the Islamic Republic of Iran, abortion is considered a crime and is punishable if performed without a valid excuse or legal permission.

For this reason, and since there are no specialized pregnancy clinics and counseling programs in Iran, primarily people who had overcome the crisis to some extent and wanted to continue the pregnancy participated in this study. It seems that crisis counseling would be more effective in reducing anxiety when it is provided in the early days of finding out about a critical pregnancy. In addition, it is suggested that in future studies, this study be replicated in laboratories where women get informed of an unwanted pregnancy.

## 5. Conclusion

Unplanned pregnancy causes women severe anxiety, doubt, and hesitation, such that they experience personal crises and struggle to get through them as soon as possible. This study shows that the crisis counseling technique reduces overt and covert anxiety. Therefore, establishing critical pregnancy clinics with midwifery consultants to assess women′s anxiety and provide information will be effective and can be considered a form of social support.

**Nomenclature**
GHQ: general health questionnaire
STAI: State–Trait Anxiety Inventory
MCC: medical crisis counseling
LBW: low birth weight

## Consent

A signed consent form from the study participant is retained on file.

## Disclosure

The present article is part of a master′s thesis on counseling in midwifery approved by Mazandaran University of Medical Sciences with the registration code 3181. All authors read and approved the final manuscript.

## Conflicts of Interest

The authors declare no conflicts of interest.

## Author Contributions

Sabura Faqhani helped design the study and intervention, oversaw analyses and the interpretation of results, content creation and review, data architecture schema, and engaged in both writing and critical review of the manuscript. Forouzan Elyasi participated in the study and intervention design, provided some resources, was part of the clinical team, visited the referred participant, and edited the manuscript. Seyed Abolhassan Naqibi helped search in the database, contributed to the design of the intervention and study, and critical revision of the manuscript. Seyed Nouroldin Mousavi Nasab assisted with the design, revised code for data cleaning and analysis of the necessary data extraction, and critical review of the manuscript. Mohammad Geran participated in the design and implementation of the study and editing of the manuscript. Soghra Khani helped design the research and intervention, oversaw analyses and the interpretation of results, and critically reviewed the manuscript.

## Funding

This work was supported by Mazandaran University of Medical Sciences (10.13039/501100004160; 3181).

## Data Availability

The data that support the findings of this study are available from the corresponding author upon reasonable request.
